# Bioabsorbable mesh use in midline abdominal wall prophylaxis and repair achieving fascial closure: a cross-sectional review of stage of innovation

**DOI:** 10.1007/s10029-020-02217-3

**Published:** 2020-05-24

**Authors:** S. K. Kamarajah, N. J. Smart, I. R. Daniels, T. D. Pinkney, R. L. Harries

**Affiliations:** 1 Department of Hepatobiliary and Pancreatic Surgery, Newcastle University NHS Trust Hospitals, Newcastle, UK; 2 Institute of Cellular Medicine, Newcastle University, Newcastle, UK; 3 Exeter Surgical Health Services Research Unit (HeSRU), Royal Devon and Exeter Hospital, Exeter, Devon UK; 4 Academic Department of Surgery, University of Birmingham, Birmingham, UK; 5 Department of Colorectal Surgery, Morriston Hospital, Swansea, SA6 6NL UK

**Keywords:** Incisional hernia, Prophylaxis, Bioabsorbable mesh, Biosynthetic mesh

## Abstract

**Background:**

Achieving stable closure of complex or contaminated abdominal wall incisions remains challenging. This study aimed to characterise the stage of innovation for bioabsorbable mesh devices used during both midline closure prophylaxis and complex abdominal wall reconstruction and to evaluate the quality of current evidence.

**Methods:**

A systematic review of published and ongoing studies was performed until 31st December 2019. Inclusion criteria were studies where bioabsorbable mesh was used to support fascial closure either prophylactically after midline laparotomy or for repair of incisional hernia with midline incision. Exclusion criteria were: (1) study design was a systematic review, meta-analysis, letter, review, comment, or conference abstract; (2) included less than *p* patients; (3) only evaluated biological, synthetic or composite meshes. The primary outcome measure was the IDEAL framework stage of innovation. The key secondary outcome measure was the risk of bias in non-randomised studies of interventions (ROBINS-I) criteria for study quality.

**Results:**

Twelve studies including 1287 patients were included. Three studies considered mesh prophylaxis and nine studies considered hernia repair. There were only two published studies of IDEAL 2B. The remainder was IDEAL 2A studies. The quality of the evidence was categorised as having a risk of bias of a moderate, serious or critical level in nine of the twelve included studies using the ROBINS-I tool.

**Conclusion:**

The evidence base for bioabsorbable mesh is limited. Better reporting and quality control of surgical techniques are needed. Although new trial results over the next decade will improve the evidence base, more trials in emergency and contaminated settings are required to establish the limits of indication.

## Introduction

Incisional hernias occur commonly after midline abdominal surgery, with a reported incidence of 12.8% at 2 years follow-up in a systematic review of 14,618 patients [1]. Incisional hernias can result in a reduced quality of life, carry a risk of strangulation, and present a significant financial burden for the health service [2].

There has been significant interest in both the prevention and repair of incisional hernia with the use of mesh. In 2010, the Ventral Hernia Working Group (VHWG) recommended against the use of permanent synthetic mesh for both Grade 3 and Grade 4 ventral hernias (contaminated or infected) and suggested that there was an increased risk of surgical site occurrence with the use of permanent synthetic mesh in Grade 2 ventral hernias (co-morbid e.g. smoker, obese, diabetic, immunocompromised), and, therefore, the choice of biological mesh may pose an advantage in such cases [3]. However, in recent years, there has been the development of bioabsorbable meshes (delayed absorbable synthetic) as a possible cost-effective alternative to biological meshes. In clinical practice, mesh choice can pose a dilemma to the surgeon; balancing both the advantage of recurrence prevention against the risk of mesh complications for the patient, whilst also considering the cost implications for the health service.

This review aimed to determine the quality and stage of innovation of the evidence supporting the use of bioabsorbable mesh in both prophylaxis after midline fascial closure and during abdominal wall reconstruction with primary midline fascial closure.

## Methods

### Search strategy

A systematic search of PubMed, EMBASE, and the Cochrane Library until 31st December 2019 was performed by two independent investigators (SKK, RH). The ClinicalTrials.gov database was also queried for ongoing studies. The search terms used were “biosynthetic mesh” or “bioabsorbable mesh”, and “ventral hernia”, “incisional hernia,” or “abdominal wall reconstruction”, individually or in combination. The “related articles’’ function was used to broaden the search, and all citations were considered for relevance. A manual search of reference lists in recent reviews and eligible studies was also undertaken. This manuscript is reported according to the preferred reporting items for systematic reviews and meta-analysis (PRISMA) [4] and assessing the methodological quality of systematic reviews (AMSTAR-2) [5] guidelines. This study was prospectively registered with the PROSPERO database (Registration CRD42020160307).

### Inclusion/exclusion criteria

Studies were included according to the following criteria: (1) evaluated use of a bioabsorbable mesh to support primary fascial closure of midline abdominal wounds or repair of incisional hernia with midline incision; (2) study design was a randomized controlled trial (RCT), prospective observational study, retrospective cohort study, or case series; (3) only included patients aged 16 years and older; (4) articles published in English only. Studies were excluded according to the following criteria: (1) study design was a systematic review, meta-analysis, letter, review, comment, or conference abstract; (2) included less than three patients; (3) only evaluated biological, synthetic, or composite meshes.

### Study outcome measures

The primary outcome measure was the stage of innovation, according to the idea, development, exploration, assessment, long-term follow-up (IDEAL) framework [6]. The level of evidence in the IDEAL staging system were 1 (case series with high risk of bias), 2A (prospective development study), 2B (research database; explanatory or feasibility RCT; prospective exploration study), 3 (RCT), and 4 (high-quality prospective registries with long-term monitoring and low risk of bias). All assessments within this study were carried out independently by two authors (SKK and RLH) and disagreement was resolved by re-examining the relevant article until consensus was achieved (NJS).

### Secondary outcome measures

One secondary outcome measure was the quality of evidence assessed using the risk of bias in non-randomised studies of interventions (ROBINS-I) [7]. The ROBINS-I views each study as an attempt to emulate (mimic) a hypothetical pragmatic randomised trial and covers seven distinct domains through which bias might be introduced. These domains are divided according to pre-intervention (bias due to confounding and bias in selection of participants into the study), at intervention (bias in classification of interventions), and post-intervention (bias due to deviations from intended interventions, bias due to missing data, bias in measurement of outcomes, and bias in selection of the reported result). Each of these seven domains is graded according to low, moderate, critical, serious or no information. The other secondary outcome measures of interest were the number of studies reporting (1) outcomes according to the European Hernia Society (EHS) consensus statement [8], (2) methodology reported according to strengthening the reporting of cohort studies in surgery (STROCCS) [9] for observational studies or consolidated standards of reporting trials (CONSORT) [10] for randomised clinical trials (3) incidence of incisional hernia (in prophylaxis studies) or recurrence (in hernia repair studies), (4) surgical site infection (SSI) rate, (5) seroma rate, and (6) mesh explantation rate.

### Data extraction

Patient demographics, indications, and type of bioabsorbable mesh used were extracted. Studies were grouped into those examining prophylactic placement in primary closure of laparotomy only (‘prophylaxis’), repair of incisional hernia/complex abdominal wall reconstruction only (‘repair’) or reporting both (‘mixed’). Descriptions of procedures performed were collected including surgical technique, number of procedures previously performed by the surgeon and monitoring of technique. Degree of contamination (clean contaminated, contaminated, and dirty surgery) was defined according to the Centre for Disease Control Surgical Wounds Classification [11] and location of bioabsorbable mesh placement was also evaluated: intraperitoneal, preperitoneal, retro-muscular, retro-rectus, inlay, or onlay [12, 13]. Urgency of surgery (i.e. elective and emergency) was also extracted from each study.

### Statistical analysis

We pre-planned our analysis to be primarily descriptive in nature. We did not predict the need for modelling or multivariable analyses. Event rates are reported as percentages (%). Continuous variables were tested for normality. Normal data were presented as mean and non-normal data as median with interquartile range.

## Results

**Fig. 1:**
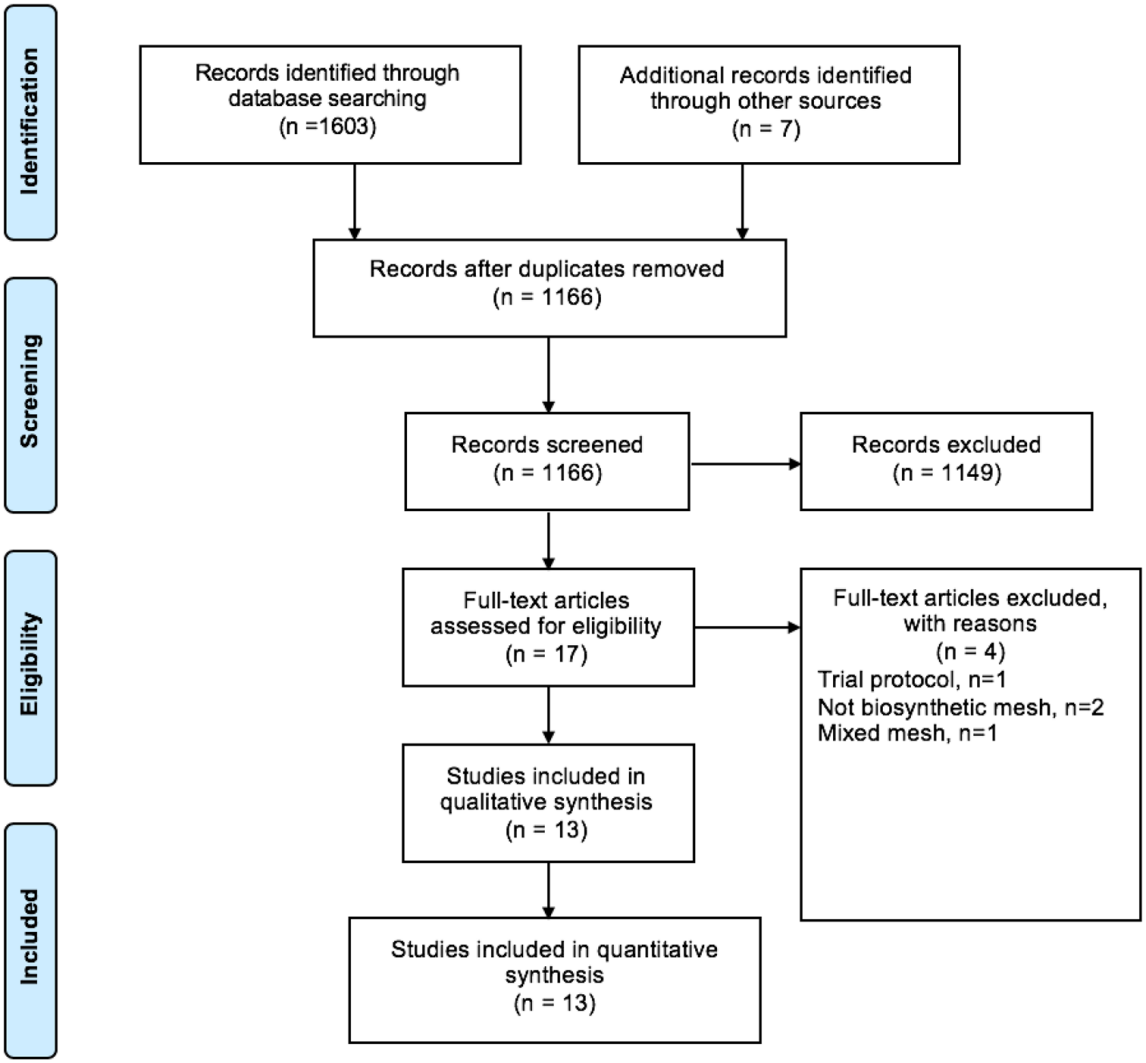
PRISMA diagram of inclusion and exclusion of studies

From 1609 studies short-listed, 12 full-text articles met the inclusion criteria (Fig. 1). Of these, three (25%) examined bioabsorbable mesh for prophylaxis [14–16] and nine (75%) reported repair after incisional hernia/abdominal wall reconstruction [17–25]. Studies for prophylaxis included a total of 201 patients with a median follow-up of 11 months (range 9–24 months) and those for repair included 1086 patients with a median follow-up of 23 months (range 1–26 months).

Tables 1 and 2 summarises the characteristics of the included studies. Gore-BioA® (WL Gore, Arizona, USA) was the most used mesh for prophylaxis of the abdominal wall used in two studies [15, 16], followed by TIGR® (Novus Scientific, Uppsala, Sweden) in one study [14]. In papers reporting bioabsorbable mesh for repair only, Phasix™ (BD Bard, Rhode Island, USA) [18, 19, 24, 25] was the most commonly reported followed by Gore-BioA® (*n* = 3) [20, 22, 23]. One study [21] reported use of Phasix™, TIGR®, Gore-BioA®, and another study [17] did not report type of bioabsorbable mesh used.

**Table 1:** Patient characteristics of included studies

Study name	Study design	Median (IQR) or mean (SD) Age, years	Patients, *n*	Patients with bioabsorbable mesh, *n*	Male, %	Median (IQR) or mean (SD) BMI kg/m^2^	Smokers, %*	Diabetes, %*	COPD, %*
Prophylaxis									
Soderback (2016) [14]	PCS	65.0 (53.5–67.5)	16	16	31	NR	38	6	13
Jordan (2018) [15]	RCS	50.4 (± 9.3)^**#**^	87	65	0	29.5 (± 5.4)^**#**^	5	9	NR
Pizza (2019) [16]	RCT	58.0 (29.0–88.0)	100	50	NR	28.0 (17.0–35.0)	26	16	30
Repair									
Cobb (2015)*** [17]	RCS	NR	255	35	NR	32.2 (15.0–66.6)	NR	NR	NR
Buell (2016) [18]	RCS	56.9	73	31	74	29.9	3	26	NR
Plymale (2017) [19]	PCS	52.0 (44.0–62.0)	31	31	45	33.0 (28.6–38.4)	23	26	29
Rosen (2017) [20]	PCS	58.0 (27.0–91.0)	104	104	40	28.0 (17.0–40.0)	19	18	11
Sahoo (2017) [21]	PCS	61.0 (51.0–70.0)	232	58	NR	31.0 (27.0–38.0)	7	24	NR
Cho (2018) [22]	RCS	56.5 (± 11.7)^**#**^	81	81	33	34.9 (± 8.4)^**#**^	11	28	NR
Garcia-Urena (2018) [23]	PCS	60.9 (32.0–86.0)	169	169	59	30.7 (20.3–46.9)	36	31	16
Roth (2018) [24]	PCS	54.7 (± 12.0)^**#**^	121	121	38	32.2 (± 4.5)^**#**^	23	33	28
Pakula (2019) [25]	RCS	47.0 (± 13.0)^**#**^	20	20	50	35.0 (± 7.4)^**#**^	NR	35	NR

*ASA* American Society of Anesthsiologists, *BMI* body mass index, *CVD* cardiovascular disease, *COPD* chronic obstructive pulmonary disease, *HTN* hypertension, *IQR* interquartile range, *n* numbers, *NR* not reported, *PCS* prospective cohort study, *RCT* randomised controlled trial, *RCS* retrospective cohort study, *SD* standard deviation

*Refers to patients in the mesh group only

**Reported as 40% for CVD and hypertension combined

***No baseline patient characteristics reported for bioabsorbable mesh

^#^Where indicated, data presented as mean and standard deviation

**Table 2:** Summary of surgery and mesh characteristics (arranged chronologically by indication)

Study name	Study country	Type of bioabsorbable mesh	Mesh placement	Fascial closure suture and technique	Hernia size, cm^2^	CDC class			
						Grade I, %	Grade II, %	Grade III, %	Grade V, %
Prophylaxis									
Soderback (2016) [14]	Sweden	TIGR®	Onlay	Continuous PDS®	NA	NR	NR	NR	NR
Jordan (2018) [15]	USA	Gore Bio-A®	Retro-rectus	Interrupted	NA	NR	NR	NR	NR
Pizza (2019) [16]	Italy	Gore Bio-A®	Retro-rectus	Continuous PDS® (post sheath) Continuous PDS® (ant sheath)	NA	0	56	44	0
Repair									
Cobb (2015) [17]	USA	NR	Retro-rectus	Continuous 2/0 PDS® (post sheath) 0 PDS® (ant sheath)	NR*	1	NR***	NR***	NR***
Buell (2016) [18]	USA	Phasix™	Mixed	NR	NR	NR	NR	NR	NR
Plymale (2017) [19]	USA	Phasix™	Retro-rectus	Absorbable (post sheath) PDS® (ant sheath)	105 (74–130)	97	3	0	0
Rosen (2017) [20]	USA	Gore Bio-A®	Mixed	NR	137 (10–513)	0	23	77	0
Sahoo (2017) [21]	USA	Gore Bio-A®, Phasix™, TIGR®	Mixed	NR	10 (7–15)	0	59	41	0
Cho (2018) [22]	USA	Gore Bio-A®	Mixed (Retro-rectus versus intraperitoneal)	Continuous 0 PDS® (post sheath) Continuous 1 PDS® (ant sheath)	148 (± 123)	68	17	1	14
Garcia-Urena (2018) [23]	Spain	Gore Bio-A®**	Retro-rectus	Continuous absorbable monofilament (post sheath) PDS® (ant sheath)	447 (240–1380)	85	7	7	2
Roth (2018) [24]	USA	Phasix™	Mixed	NR	116 (± 81)	100	0	0	0
Pakula (2019) [25]	USA	Phasix™	Retro-rectus	Continuous 2/0 PDS® (post sheath) 0 Prolene® or PDS® (ant sheath)	533 (± 500)	35	25	40	0

*NA* not applicable, *NR* not reported, *PDS* polydioxanone, *USA* United States of America

*Hernia size and median follow-up not reported specifically for those who had bioabsorbable mesh

**Combined use with a polypropylene mesh

***Reported as CDC Class II–V = 33

### IDEAL stage of innovation and ROBINS-I quality of evidence

Distribution of IDEAL stage and ROBINS-I of included studies are presented in Tables 3 and 4 respectively. From the three prophylaxis studies, two [14, 16] evaluated bioabsorbable mesh following midline laparotomy and one [15] following prophylactic reinforcement of abdominal wall donor site after breast reconstruction. Of these three studies, one study [15] included elective patients and another included both elective and emergency patients [16]. Another study [14] did not report surgical urgency of these included patients. The degrees of contamination were only reported in the one study [16]. Gore-BioA® was used in two studies with a retro-rectus placement [15, 16]. The other used TIGR™ in an onlay placement [14]. Two studies were IDEAL stage 2A [14, 15] and one was IDEAL stage 2B [16]. None of the included studies of prophylaxis were reported according to the STROCCS statement for observational studies.

**Table 3:** Distribution of IDEAL stage of innovation, shown by indication

Indication	IDEAL stage				
	1 (Case report)	2A (Cohort study)	2B (Feasibility RCT/research database/prospective exploration study)	3 (RCT)	4 (Registry)
Prophylaxis	0	2	1	0	0
Repair	0	8	1	0	0
Total	0	10	2	0	0

**Table 4:** Distribution of ROBINS-I of study quality, shown by indication

Study name	Baseline confounding	Selection of participants	Classification of interventions	Deviation from intended interventions	Missing data	Measurement of outcomes	Selection of reported results	Overall risk of bias
Prophylaxis								
Soderback (2016) [14]	Critical	Moderate	Serious	NI	Low	Moderate	Moderate	Critical
Jordan (2018) [15]	Serious	Moderate	Moderate	NI	Low	Moderate	Moderate	Moderate
Pizza (2019) [16]	Low	Low	Low	Low	Low	Moderate	Low	Low
Repair								
Cobb (2015) [17]	Serious	Serious	Moderate	NI	Low	Moderate	Low	Moderate
Buell (2016) [18]	Critical	Moderate	Critical	NI	Moderate	Moderate	Moderate	Moderate
Plymale (2017) [19]	Serious	Moderate	Moderate	NI	Low	Moderate	Moderate	Moderate
Rosen (2017) [20]	Moderate	Moderate	Low	NI	Low	Low	Moderate	Low
Sahoo (2017) [21]	Low	Low	Low	NI	Low	Low	Low	Low
Cho (2018) [22]	Critical	Moderate	Low	NI	Moderate	Moderate	Low	Moderate
Garcia-Urena (2018) [23]	Moderate	Moderate	Low	NI	Low	Low	Moderate	Moderate
Roth (2018) [24]	Critical	Moderate	Moderate	NI	Low	Moderate	Moderate	Moderate
Pakula (2019) [25]	Serious	Critical	Critical	NI	Low	Moderate	Moderate	Serious

From nine studies of repair, seven studies reported only elective patients undergoing repair of ventral hernia and two studies for abdominal wall reconstruction. The degree of contamination was reported in seven studies, [19–25] of which one study [24] was Centre for Centers for Disease Control and Prevention (CDC) Class I and the rest included a mixture of CDC Class II–IV procedures. Mesh placement was reported as retro-rectus in four studies [19, 22, 23, 25] and a combination in five studies [18, 20–22, 24]. Eight studies [17–19, 21–25] were IDEAL Stage 2A and one [20] was IDEAL Stage 2B. None reported standardisation of technique or location of bioabsorbable mesh placement; the choice of mesh type was based on the preference of operating surgeon. None of the included studies of repair were reported according to the STROCCS statement for observational studies.

### Outcome reporting

None of the studies in this review have reported outcomes according to the European Hernia Society (EHS) consensus statement [8]. Only one study reported ‘freedom of recurrence’ survival times [14]. In the prophylaxis group (which included three studies), one study reported a definition used for detection of incisional hernia, which included a combination of clinical examination and radiological assessment with ultrasound [12]. In the repair group (which included nine studies), five reported a definition for recurrence of hernia (clinical only [14, 19], clinical combined with computed tomography (CT) [15, 17], and clinical combined with either CT or magnetic resonance imaging [20]). In the prophylaxis group, the rate of incisional hernia was 12.2% (16/131) at a median follow-up of 24 months, whereas in the repair group, the rate of recurrence was 7.7% (43/557) with a median follow-up of 22 months.

Outcome reporting per study is described in Table 5. SSI rates were reported in two of three studies from the prophylaxis group, and seven of the nine studies in the repair group. The SSI rate was 1.5% (4/66) and 12.2% (71/584) for the prophylaxis and repair studies, respectively. Seroma rates were reported in two prophylaxis studies and six repair studies. The seroma rate was 4.5% (3/66) and 9.3% (47/503) for the prophylaxis and repair studies, respectively. Mesh explantation rates were reported in three prophylaxis studies and five repair studies. The mesh explantation rate was 0% (0/131) and 0.7% (3/443) for the prophylaxis and repair studies, respectively.

**Table 5:** Incidence of post-operative outcomes

Study name	Patients with bioabsorbable mesh, *n*	Type of bioabsorbable mesh	Incisional hernia or recurrence, %	SSI, %	Seroma, %	Mesh explantation, %	Median follow-up, months
Prophylaxis							
Soderback (2016) [14]	16	TIGR®	0	6.3	6.3	0	9
Jordan (2018) [15]	65	Gore-BioA®	20.0	NR	NR	0	11
Pizza (2019) [16]	50	Gore-BioA®	6.0	6.0	4.0	0	24
Repair							
Cobb (2015) [17]	35	NR	17.1	NR*	NR*	NR*	17
Buell (2016) [18]	31	Phasix™	6.5	NR	NR	NR	NR
Plymale (2017) [19]	31	Phasix™	0	0	12.9	0	24
Rosen (2017) [20]	104	Gore-BioA™	16.6	19.8	6.2	1.0	NR
Sahoo (2017) [21]	58	Gore-BioA®, Phasix™, TIGR®	NR	22.4	1.7	1.7	22
Cho (2018) [22]	81	Gore-BioA®	11.1	6.2	NR	0	24
Garcia-Urena (2018) [23]	169	Gore-BioA®**	2.9	12.4	19.5	0.6	26
Roth (2018) [24]	121	Phasix™	9.0	9.0	6.0	NR	NR
Pakula (2019) [25]	20	Phasix™	0	10.0	10.0	NR	21

*Outcomes not reported specifically for those who had bioabsorbable mesh

### Reporting of surgical technique

Of the 12 studies, 9 studies (75%) provided details of surgical procedure; all three studies from the prophylaxis group and six from the repair group. None of the papers reported the minimum number of procedures performed by the operating surgeons as a requirement. Suture choice was described in seven of the studies [14, 16, 17, 19, 22, 23, 25] (Table 2), however only one study described needle size used [16]. Fascial closure technique (continuous or interrupted) was described in seven of the studies [14–17, 22, 23, 25]; however, no studies mentioned suture bite size utilised (e.g. small bite versus large bite), and only two study stated that a suture length: wound length (SL:WL) ratio of > 4:1 was used; however, it was not formally recorded [14, 16].

## Discussion

This systematic review found that the evidence base for the use of bioabsorbable mesh in both prophylaxis following midline closure and repair of ventral hernias is limited. Most of the included studies were IDEAL stage 2A, with only two studies of IDEAL Stage 2B. The quality of the evidence was categorised as having a risk of bias of a moderate, serious or critical level in nine of the twelve included studies using the ROBINS-I tool.

In the included prophylaxis studies, the incisional hernia rate was found to be 12.2%, with a seroma rate of 4.5%. When examining the role of bioabsorbable mesh in prophylaxis, it should be compared to synthetic mesh. The EHS guidelines [26], published in 2015, made a weak recommendation in favour of prophylactic mesh augmentation in elective midline laparotomy in high-risk patients to prevent incisional hernia formation; however, could not make any recommendation about mesh position, fixation or mesh type due to lack of evidence. Since then, a meta-analysis of RCTs [27] demonstrated synthetic mesh had a significantly lower rates of incisional hernia (4.2% vs. 28.3%, *p* < 0.001) than the suture group, but higher seroma rates (14.6% vs. 8.9%, *p* = 0.04). Whilst the evidence to support the use of bioabsorbable mesh in prophylaxis is limited, it may be that it has a role in a sub-group of patients when wanting to avoid seroma formation, accepting a potentially higher rate of hernia formation. However, more research is required to determine this further.

Adequate length of follow-up for detection of long-term outcomes such as recurrence is critical when conducting mesh related studies. The included studies had median follow-up durations of 11 months (range 9–24 months) and 22 months (range 1–24 months) for prophylaxis and repair, respectively. Previous work has shown that the prevalence of incisional hernias after midline incisions was 12.8% at a weighted mean of 23.7 months [1], and, therefore, follow-up shorter than 2 years in abdominal wall studies is likely to underestimate the recurrence rate. The incidence of hernia recurrence in the repair group was 7.7% (range 0–17.0%), which is lower than expected. The reasons for this may in part be explained by the fact that three of the studies failed to report length of follow-up [18, 20, 24], a further study did not report recurrence rate, was reporting 30-day outcomes only [21] and five studies failed to report a definition for hernia recurrence detection [14, 15, 17, 19, 20]. Similarly, in the prophylaxis group, one study investigated endpoints of dehiscence, SSI, seroma, and pain only [14].

In 2013, the European Hernia Society (EHS) published recommendations for reporting outcomes results in abdominal wall repair research [8]. This included the reporting of the surgical technique, mesh placement and fixation method, hernia size, method of follow-up, and detection method of recurrence. It also recommended a preferred method to report the outcome of recurrence of time to event analysis using Kaplan–Meier estimates of ‘freedom of recurrence’. Few of the included studies met these criteria for reporting outcomes. Two repair studies failed to describe the median hernia size [16, 22]. There were differences in the detection of recurrence methods employed (clinical versus radiological (ultrasound versus cross-sectional imaging e.g. computed tomography) versus combination), although five studies failed to describe the detection method used [11, 13, 16, 18, 22]. Several studies included a mixture of mesh placements within their cohorts making it difficult to draw meaningful conclusions from the results. Only one study reported the outcome of recurrence using 'freedom of recurrence’ survival curves [14].

We have examined all studies using a range of different bioabsorbable meshes. It should be noted that each consist of different scaffold fibres which equate to differences in their absorption time. Gore Bio-A® consists of Polyglycolic acid and trimethylene carbonate and is resorbed within 6–7 months [28]. Phasix™ consists of poly-4-hydroxybutyrate (P4HB) and is resorbed within 12 months [29]. TIGR® consists of a fast-degrading copolymer between glycolide and trimethylene carbonate and a slow-degrading copolymer between lactic and trimethylene carbonate [30]. The fast-degrading copolymer is absorbed by 4 months, whereas the slow-degrading copolymer loses strength after 6–9 months and is completely resorbed at 3 years. Clearly, the differences in absorption time may equate to differences in outcomes such as hernia occurrence/recurrence.

Current ongoing work investigating the role of bioabsorbable mesh in abdominal wall surgery includes four observational studies and two randomised controlled trials registered on trials registries. A multicentre observational study (NCT04132986) of bioabsorbable mesh in contaminated ventral hernia repairs is currently recruiting in France and aiming to recruit 200 patients [31]. The ATLAS trial (NCT02712398) is a prospective multicentre observational study assessing the use of Phasix™ in at-risk patients (one or more co-morbidities) having a laparoscopic ventral or incisional hernia repair, aiming to recruit 120 participants [32]. Two further multicentre observational studies (NCT01961687 [33] and NCT02720042 [34]) are assessing the role of Phasix™ for ventral or incisional hernia repair. The PREBIOUS trial (NCT02208557) is a multicentre randomised controlled trial of prophylaxis in midline laparotomy closure with a bioabsorbable mesh versus suture closure only and is also currently recruiting [35]. A further study is randomising at-risk patients to either biological [Strattice™ (Allergan, Dublin, Ireland)] or bioabsorbable (Gore Bio-A®) mesh during ventral hernia repair in those with VHWG Grade 2 or Grade 3 (NCT01794338) [36]; this study is of particular interest as it directly compares bioabsorbable to biological mesh for use in repair in at-risk patient, which is likely where bioabsorbable mesh would have a key role, if outcomes are similar, due to a potential cost saving. It is hoped that the results of these ongoing studies will add to the evidence base, addressing some of the deficits in the evidence to date.

There are limitations of this study, the use of IDEAL collaboration grading can be subjective [5]; however, this was mitigated by assessment by two independent assessors. It also categorises research databases, feasibility non-randomised studies alongside randomised feasibility and pilot studies and are linked by their aims and purpose rather than study design, which could be viewed as a weakness. ROBINS-I is a tool focusing specifically on risk of bias in non-randomised trials [6] and is, therefore, not the optimal test to assess bias risk in the included feasibility RCT [12].

In summary, the evidence base for the use of bioabsorbable mesh is limited for both the prophylaxis of midline closure and the repair of abdominal wall hernias. Further studies investigating the use of bioabsorbable mesh in abdominal wall surgery are currently ongoing. Future work should report according to the European Hernia Society (EHS) published recommendations for reporting outcomes results in abdominal wall repair research [7].

## References

[1] Bosanquet DC, Ansell J, Abdelrahman T, Cornish J, Harries R, Stimpson A, Davies L, Glasbey JC, Frewer KA, Frewer NC, Russell D, Russell I, Torkington J (2015). Systematic review and meta-regression of factors affecting midline incisional hernia rates: analysis of 14,618 patients. PLoS ONE.

[2] Fischer JP, Basta MN, Mirzabeigi MN, Bauder AR, Fox JP, Drebin JA, Serletti JM, Kovach SJ (2016). A risk model and cost analysis of incisional hernia after elective, abdominal surgery based upon 12,373 cases: the case for targeted prophylactic intervention. Ann Surg.

[3] Breuing K, Butler CE, Ferzoco S, Franz M, Hultman CS, Kilbridge JF, Rosen M, Silverman RP, Vargo D (2010). Incisional ventral hernias: review of the literature and recommendations regarding the grading and technique of repair. Surgery.

[4] Liberati A, Altman DG, Tetzlaff J, Mulrow C, Gotzsche PC, Ioannidis JP, Clarke M, Devereaux PJ, Kleijnen J, Moher D (2009). The PRISMA statement for reporting systematic reviews and meta-analyses of studies that evaluate healthcare interventions: explanation and elaboration. BMJ.

[5] Shea BJ, Reeves BC, Wells G, Thuku M, Hamel C, Moran J, Moher D, Tugwell P, Welch V, Kristjansson E, Henry DA (2017). AMSTAR 2: a critical appraisal tool for systematic reviews that include randomised or non-randomised studies of healthcare interventions, or both. BMJ.

[6] McCulloch P, Altman DG, Campbell WB, Flum DR, Glasziou P, Marshall JC, Nicholl J, Aronson JK, Barkun JS, Blazeby JM, Boutron IC, Clavien PA, Cook JA, Ergina PL, Feldman LS, Maddern GJ, Reeves BC, Seiler CM, Strasberg SM, Meakins JL, Ashby D, Black N, Bunker J, Burton M, Campbell M, Chalkidou K, Chalmers I, de Leval M, Deeks J, Grant A, Gray M, Greenhalgh R, Jenicek M, Kehoe S, Lilford R, Littlejohns P, Loke Y, Madhock R, McPherson K, Meakins J, Rothwell P, Summerskill B, Taggart D, Tekkis P, Thompson M, Treasure T, Trohler U, Vandenbroucke J (2009). No surgical innovation without evaluation: the IDEAL recommendations. Lancet.

[7] Sterne JA, Hernan MA, Reeves BC, Savovic J, Berkman ND, Viswanathan M, Henry D, Altman DG, Ansari MT, Boutron I, Carpenter JR, Chan AW, Churchill R, Deeks JJ, Hrobjartsson A, Kirkham J, Juni P, Loke YK, Pigott TD, Ramsay CR, Regidor D, Rothstein HR, Sandhu L, Santaguida PL, Schunemann HJ, Shea B, Shrier I, Tugwell P, Turner L, Valentine JC, Waddington H, Waters E, Wells GA, Whiting PF, Higgins JP (2016). ROBINS-I: a tool for assessing risk of bias in non-randomised studies of interventions. BMJ.

[8] Muysoms FE, Deerenberg EB, Peeters E, Agresta F, Berrevoet F, Campanelli G, Ceelen W, Champault GG, Corcione F, Cuccurullo D, DeBeaux AC, Dietz UA, Fitzgibbons RJ, Gillion JF, Hilgers RD, Jeekel J, Kyle-Leinhase I, Kockerling F, Mandala V, Montgomery A, Morales-Conde S, Simmermacher RK, Schumpelick V, Smietanski M, Walgenbach M, Miserez M (2013). Recommendations for reporting outcome results in abdominal wall repair: results of a Consensus meeting in Palermo, Italy. Hernia.

[9] Agha RA, Borrelli MR, Vella-Baldacchino M, Thavayogan R, Orgill DP (2017). The STROCSS statement: strengthening the reporting of cohort studies in surgery. Int J Surg.

[10] Schulz KF, Altman DG, Moher D (2010). CONSORT 2010 statement: updated guidelines for reporting parallel group randomised trials. BMJ.

[11] Mangram AJ, Horan TC, Pearson ML, Silver LC, Jarvis WR (1999). Guideline for prevention of surgical site infection, 1999. Centers for Disease Control and Prevention (CDC) Hospital Infection Control Practices Advisory Committee. Am J Infect Control.

[12] Muysoms F, Jacob B (2017) International hernia collaboration consensus on nomenclature of abdominal wall hernia repair. In World J Surg, United States, 2017. 10.1007/s00268-017-4115-3 28717915

[13] Parker SG, Halligan S, Erotocritou M, Wood CPJ, Boulton RW, Plumb AAO, Windsor ACJ, Mallett S (2019). A systematic methodological review of non-randomised interventional studies of elective ventral hernia repair: clear definitions and a standardised minimum dataset are needed. Hernia.

[14] Soderback H, Mahteme H, Hellman P, Sandblom G (2016). Prophylactic resorbable synthetic mesh to prevent wound dehiscence and incisional hernia in high high-risk laparotomy: a pilot study of using TIGR matrix mesh. Front Surg.

[15] Jordan SW, Schulz SA, Carraher AM, Cabiling DS (2019). Comparison of polypropylene and bioabsorbable mesh for abdominal wall reinforcement following microsurgical breast reconstruction. J Reconstr Microsurg.

[16] Pizza F, D’Antonio D, Arcopinto M, Dell’Isola C, Marvaso A (2019) Safety and efficacy of prophylactic resorbable biosynthetic mesh following midline laparotomy in clean/contemned field: preliminary results of a randomized double blind prospective trial. Hernia 2019. 10.1007/s10029-019-02025-4 31432287

[17] Cobb WS, Warren JA, Ewing JA, Burnikel A, Merchant M, Carbonell AM (2015). Open retromuscular mesh repair of complex incisional hernia: predictors of wound events and recurrence. J Am Coll Surg.

[18] Buell JF, Sigmon D, Ducoin C, Shapiro M, Teja N, Wynter E, Hanisee MK, Parker G, Kandil E, Darden M (2017). Initial experience with biologic polymer scaffold (poly-4-hydroxybuturate) in complex abdominal wall reconstruction. Ann Surg.

[19] Plymale MA, Davenport DL, Dugan A, Zachem A, Roth JS (2018). Ventral hernia repair with poly-4-hydroxybutyrate mesh. Surg Endosc.

[20] Rosen MJ, Bauer JJ, Harmaty M, Carbonell AM, Cobb WS, Matthews B, Goldblatt MI, Selzer DJ, Poulose BK, Hansson BM, Rosman C, Chao JJ, Jacobsen GR (2017). Multicenter, prospective, longitudinal study of the recurrence, surgical site infection, and quality of life after contaminated ventral hernia repair using biosynthetic absorbable mesh: the COBRA study. Ann Surg.

[21] Sahoo S, Haskins IN, Huang LC, Krpata DM, Derwin KA, Poulose BK, Rosen MJ (2017). Early wound morbidity after open ventral hernia repair with biosynthetic or polypropylene mesh. J Am Coll Surg.

[22] Cho JE, Helm MC, Helm JH, Mier N, Kastenmeier AS, Gould JC, Goldblatt MI (2019). Retro-rectus placement of bio-absorbable mesh improves patient outcomes. Surg Endosc.

[23] Garcia-Urena MA, Lopez-Monclus J, Cuccurullo D, Blazquez Hernando LA, Garcia-Pastor P, Reggio S, Cubedo EJ, Méndez CSM, Cidoncha AC, de Lersundi ARV (2019). Abdominal wall reconstruction utilizing the combination of absorbable and permanent mesh in a retromuscular position: a multicenter prospective study. World J Surg.

[24] Roth JS, Anthone GJ, Selzer DJ, Poulose BK, Bittner JG, Hope WW, Dunn RM, Martindale RG, Goldblatt MI, Earle DB, Romanelli JR, Mancini GJ, Greenberg JA, Linn JG, Parra-Davila E, Sandler BJ, Deeken CR, Voeller GR (2018). Prospective evaluation of poly-4-hydroxybutyrate mesh in CDC class I/high-risk ventral and incisional hernia repair: 18-month follow-up. Surg Endosc.

[25] Pakula A, Skinner R (2020). Outcomes of open complex ventral hernia repairs with retromuscular placement of poly-4-hydroxybutyrate bioabsorbable mesh. Surg Innov.

[26] Muysoms FE, Antoniou SA, Bury K, Campanelli G, Conze J, Cuccurullo D, de Beaux AC, Deerenberg EB, East B, Fortelny RH, Gillion JF, Henriksen NA, Israelsson L, Jairam A, Janes A, Jeekel J, Lopez-Cano M, Miserez M, Morales-Conde S, Sanders DL, Simons MP, Smietanski M, Venclauskas L, Berrevoet F (2015). European Hernia Society guidelines on the closure of abdominal wall incisions. Hernia.

[27] Payne R, Aldwinckle J, Ward S (2017). Meta-analysis of randomised trials comparing the use of prophylactic mesh to standard midline closure in the reduction of incisional herniae. Hernia.

[28] Gore Medical (2020). Gore Bio-A^®^ tissue reinforcement. https://www.goremedical.com/products/bioatissue. Accessed 7 Apr 2020.

[29] Bard BD (2020) Phasix^TM^ mesh. https://www.crbard.com/Davol/en-US/products/Phasix-Mesh. Accessed 7 Apr 2020.

[30] Novus Scientific (2020). TIGR^®^ Matrix. https://novusscientific.com/row/products/tigr-matrix/technology/. Accessed 7 Apr 2020.

[31] Romain B (2019) Study of absorbable biosynthetic meshes in contaminated ventral hernia repair. 2019. https://clinicaltrials.gov/ct2/show/NCT04132986?term=biosynthetic+mesh&draw=2&rank=1. Accessed 27 Jan 2020.

[32] Bard C (2016) A prospective trial of a bio-absorbable mesh in challenging laparoscopic ventral or incisional hernia repair (ATLAS). 2016. https://clinicaltrials.gov/ct2/show/NCT02712398?term=bioabsorbable+mesh&draw=2&rank=1. Accessed 27 Jan 2020.

[33] Bard C (2013) A prospective, multi-center study of Phasix^TM^ Mesh for ventral or incisional hernia repair. 2013. https://clinicaltrials.gov/ct2/show/NCT01961687. Accessed 4 Apr 2020

[34] van Rooijen MMJ, Jairam AP, Tollens T, Jorgensen LN, de Vries Reilingh TS, Piessen G, Kockerling F, Miserez M, Windsor ACJ, Berrevoet F, Fortelny RH, Dousset B, Woeste G, van Westreenen HL, Gossetti F, Lange JF, Tetteroo GWM, Koch A, Kroese LF, Jeekel J (2018). A post-market, prospective, multi-center, single-arm clinical investigation of Phasix mesh for VHWG grade 3 midline incisional hernia repair: a research protocol. BMC Surg.

[35] Lopez-Cano M, Pereira JA, Lozoya R, Feliu X, Villalobos R, Navarro S, Arbos MA, Armengol-Carrasco M (2014). PREBIOUS trial: a multicenter randomized controlled trial of PREventive midline laparotomy closure with a BIOabsorbable mesh for the prevention of incisional hernia: rationale and design. Contemp Clin Trials.

[36] Heniford BT (2013) The use of biologic mesh vs bioabsorbable mesh during ventral hernia repair in at-risk patients. 2013. https://clinicaltrials.gov/ct2/show/NCT01794338?id=NCT01794338&draw=2&rank=1. Accessed 27 Jan 2020

